# Human leukocyte antigen-G isoform HLA-G2/6, but not HLA-G1/4/5, is an independent indicator of poor survival in patients with colorectal cancer

**DOI:** 10.3389/fimmu.2025.1672144

**Published:** 2025-10-21

**Authors:** Xia Zhang, Qiu-Yue Han, Jian-Gang Zhang, Jiao Lin, Wei-Hua Yan, Aifen Lin

**Affiliations:** ^1^ Biological Resource Center, Taizhou Hospital of Zhejiang Province, Wenzhou Medical University, Linhai, Zhejiang, China; ^2^ Key Laboratory of Human Leukocyte antigen - G (HLA-G) Research & Development of Taizhou, Taizhou Hospital of Zhejiang Province, Wenzhou Medical University, Linhai, Zhejiang, China; ^3^ Department of Urology, Taizhou Hospital of Zhejiang Province, Wenzhou Medical University, Linhai, Zhejiang, China; ^4^ Key Laboratory of Minimally Invasive Techniques & Rapid Rehabilitation of Digestive System Tumor of Zhejiang Province, Taizhou Hospital of Zhejiang Province, Wenzhou Medical University, Linhai, Zhejiang, China; ^5^ Medical Research Center, Taizhou Hospital of Zhejiang Province, Wenzhou Medical University, Linhai, Zhejiang, China

**Keywords:** HLA-G, isoforms, monoclonal antibody, colorectal cancer, prognosis

## Abstract

**Background:**

Human leukocyte antigen (HLA)-G has multiple isoforms with unique molecular structures and receptor-binding specificities. Different HLA-G isoform(s) may have distinct clinical relevance. Because of the lack of isoform-specific monoclonal antibodies (mAbs), the clinical significance of HLA-G isoforms (HLA-G1 to HLA-G7), except HLA-G1 and HLA-G5, remains largely unknown.

**Methods:**

In this study, mAbs against HLA-G2/6 and HLA-G1/4/5 isoforms were generated and characterized. Expression of HLA-G2/6 and HLA-G1/4/5 isoforms was analyzed by immunohistochemistry, and clinical significance was evaluated retrospectively in 345 patients with colorectal cancer (CRC).

**Results:**

The expression rate of HLA-G2/6 (90/345, 26.1%) was significantly lower than that of HLA-G1/4/5 (275/345, 79.7%; *p* < 0.001). Patients with HLA-G2/6 expression had significantly poorer overall survival (OS) (median OS: 6.3 years [95% CI: 4.1–8.5] *vs*. 10.0 years [95% CI: 7.6–12.4]; *p* = 0.008). Multivariate Cox proportional-hazard model results indicated that HLA-G2/6 was an independent prognostic factor for CRC (hazard ratio [HR] = 1.530, 95% CI: 1.125–2.081; *p* = 0.007). Moreover, HLA-G2/6 expression showed stratified prognostic significance among several CRC patient subgroups, specifically in female patients (p = 0.003), younger patients (<66 years p < 0.001), patients with colon cancer (*p* = 0.045), those at stage pT3 (*p* = 0.008), pN1 (*p* = 0.020), *p*M0 (*p* = 0.009), and AJCC stage III (*p* = 0.005). However, no statistical significance was found between HLA-G1/4/5 isoform expression and patient prognosis in CRC.

**Conclusions:**

This is the first study to generate mAbs for the HLA-G2/6 and HLA-G1/4/5 isoforms. Our findings reveal that HLA-G2/6—but not HLA-G1/4/5—expression is an independent prognostic indicator for patients with CRC. In the context of precision medicine, our study also suggests that HLA-G isoform typing may be necessary for HLA-G-targeted cancer immunotherapy.

## Introduction

With the development of various targeted therapeutic agents, substantial progress has been achieved in cancer immunotherapy following the introduction of numerous targeted therapeutic products (1). The first immune checkpoint inhibitor (ICI) against cytotoxic T-lymphocyte–associated protein 4 (CTLA-4) was approved by the FDA in 2011. Since then, other ICIs, such as programmed death protein 1/programmed cell death ligand 1 (PD-1/PD-L1) antibodies, have provided promising alternative treatments for certain types of advanced cancer, although only a few patients benefit clinically because of primary or adaptive resistance and life-threatening immune-related adverse events (irAEs), such as in patients with colorectal cancer (CRC) (2–4). To overcome the limitations of current ICI-based cancer immunotherapies, novel immune checkpoints are being explored, including the human leukocyte antigen-G (HLA-G) checkpoint.

HLA-G is a non-classical HLA class I antigen that induces immune suppression and is closely associated with poor prognosis, making it a promising non-self- and tumor-site-agnostic target for immunotherapy. HLA-G-targeted clinical trials using various strategies for multiple advanced cancers, including CRC, have been conducted since 2020 (https://clinicaltrials.gov/search?cond=HLA-G) (5).

HLA-G belongs to a subgroup of non-classical HLA class I antigens (HLA-E, HLA-F, HLA-G, and HLA-H). In contrast to the classical HLA class I antigens, which have been extensively investigated, the immune-modulatory roles and clinical significance of the non-classical HLA class I antigens—particularly HLA-G—have attracted increasing attention in cancer biology (6, 7). Unlike the classical HLA class I antigens (HLA-A, -B, and -C), which are ubiquitously expressed in nucleated cells, HLA-G expression is restricted to a limited number of tissues under physiological conditions, whereas aberrant expression is frequently observed in pathological settings (8). In cancer, tumor-specific expression of HLA-G was first observed in melanoma in 1998 (9). Since then, over the past three decades, the immunosuppressive functions and underlying mechanisms of HLA-G have been intensively explored, and its clinical significance has been evaluated in more than 30 types of cancer (8). Studies have shown that HLA-G interacts with inhibitory receptors such as immunoglobulin-like transcripts (ILT) 2 and ILT4 to inhibit immune-cell functions, including (a) antigen-presenting cell maturation; (b) natural killer (NK) cell and T-cell cytotoxicity, proliferation, and anti-tumor cytokine or chemokine production; (c) B-cell proliferation, antibody production, and chemotaxis; and (d) neutrophil phagocytosis and reactive oxygen species (ROS) production (6, 10). HLA-G also induces the proliferation of tolerogenic dendritic cells (DCs) and myeloid-derived suppressor cells (MDSCs), promotes regulatory T cells (Tregs), and polarizes M1 macrophages toward M2 cells (11). Engagement between HLA-G and killer-cell immunoglobulin-like receptor 2DL4 (KIR2DL4) desensitizes breast cancer cells to trastuzumab treatment or promotes metastasis by inducing matrix metalloproteinase (MMP)-9 expression (12, 13). Furthermore, the pro-tumorigenic significance of HLA-G has been confirmed by numerous preclinical studies using tumor-bearing mouse models, demonstrating that HLA-G can inhibit host innate and adaptive anti-tumor immune responses and consequently promote tumor metastasis and shorten survival of mice (14–16). Moreover, blockade of HLA-G with specific antibodies and the development of anti-HLA-G chimeric antigen receptor (CAR) NK or CAR-T cells have demonstrated that HLA-G is a valid target for cancer immunotherapy (17–21).

Notably, pre-mRNA alternative splicing is ubiquitous in eukaryotes, generating different isoforms from one gene with distinct molecular structures and even opposing biological functionalities (22). The imbalance or heterogeneity of pro- or anti-tumor isoforms resulting from aberrant alternative splicing during tumorigenesis and disease progression is well recognized (23, 24). In this context, at least seven α1 domain–containing isoforms (membrane-bound HLA-G1 to HLA-G4 and soluble HLA-G5 to HLA-G7) have been identified. Functionally, both membrane-bound and soluble HLA-G isoforms can impair anti-tumor immune responses and are associated with cancer progression (25, 26). Among the HLA-G isoforms, HLA-G1 and HLA-G5 contain α1, α2 and α3 domains, whereas the others lack α2 and/or α3 extracellular domains. HLA-G2 and HLA-G6 contain α1 and α3; HLA-G3 and HLA-G7 contain only the α1 domain; and HLA-G4 contains α1 and α2 domains. Because of the lack of isoform-specific antibodies, the clinical significance of individual HLA-G isoforms remains unclear (8, 27, 28). Most previous functional or mouse model analyses were based on the HLA-G1 isoform, and the clinical relevance of HLA-G expression has generally been evaluated using the widely applied monoclonal antibody (mAb) 4H84 (8). The mAb 4H84 was generated against the 61^st^–83^rd^ amino acid peptide located in the α1 domain of HLA-G. It recognizes all denatured α1 domain–containing HLA-G isoforms (HLA-G1 to HLA-G7) but cannot distinguish among individual isoforms (29, 30). Thus, a positive result with mAb 4H84 only indicates total expression of α1 domain–containing HLA-G isoforms and does not differentiate between splice variants or quantify their relative abundance in cancer lesions (28). Furthermore, intratumoral, intertumoral, and interpatient heterogeneity in HLA-G isoform expression has been frequently observed, including in CRC, raising concerns about the precision of HLA-G-targeted cancer immunotherapy (11, 28, 31, 32).

In this study, we developed mAbs against the HLA-G2/6 and HLA-G1/4/5 isoforms, respectively. Expression of HLA-G2/6 and HLA-G1/4/5 in 345 patients with colorectal cancer (CRC; *n* = 176 colon, *n* = 169 rectal) was analyzed using immunohistochemistry, and their clinical relevance was evaluated.

## Materials and methods

### Ethics statement

This study was approved by the Ethics Committee of Taizhou Hospital of Zhejiang Province, China (K20240907). Written informed consent was obtained from each patient with colorectal cancer (CRC). All animal experiments were approved by the Institutional Ethics Committee for Laboratory Animal Experimentation, Taizhou Enze Medical Center (Group) (Tab of Animal Experimental Ethical Inspection No.: tzy-2019057). Animal experiments were performed in accordance with the national legislation of China on the Guidance for the Care and Use of Laboratory Animals.

### Cloning and expression of HLA-G1~HLA-G6 isoforms

Complementary DNA (cDNA) was synthesized from total RNA extracted from JEG-3 cells (National Collection of Authenticated Cell Cultures, Shanghai, China) using TRIZOL reagent (Invitrogen, NY, USA). The primers used to amplify different HLA-G isoforms are listed in **Supplementary Table 1**. Polymerase chain reaction (PCR) products with expected sizes corresponding to the various HLA-G isoforms were excised, ligated into the pGEM^®^-T Easy vector (Promega, WI, USA), and confirmed by sequencing. K562 cells were transfected with recombinant pVITRO2-mcs vectors (Invivogen, NY, USA) containing HLA-G1–HLA-G6 cDNA using Lipofectamine 2000 reagent (Invitrogen, NY, USA) and screened with hygromycin B (Amresco, OH, USA).

Expression of HLA-G1–HLA-G6 isoforms was confirmed by Western blotting using mAb 4H84 (detecting an epitope within the α1 domain of the HLA-G heavy chain) and mAb 5A6G7 (detecting the C-terminal region of the heavy chain in the HLA-G5/HLA-G6 isoforms; Exbio, Czech Republic).

### Monoclonal antibody generation

Monoclonal antibodies (mAbs) for the HLA-G2/6 isoforms (clone YWHG-26) and HLA-G1/4/5 isoforms (clone YWHG-4) were generated by immunizing female BALB/c mice with synthetic peptides corresponding to amino acids in the junction region between α1 and α3 (RGYYNQSEAKPPKTHVTHHPV), shared specifically by HLA-G2 and HLA-G6, and between α1 and α2 (RGYYNQSEASSHTLQWMIG), shared specifically by HLA-G1, HLA-G4, and HLA-G5 (**Supplementary Figures 1A, B**). Peptide synthesis, peptide-KLH conjugation, mouse immunization, and hybridoma generation were performed by China Peptides Co., Ltd. (Shanghai, China).

Hybridoma supernatants were screened for reactivity using enzyme-linked immunosorbent assay (ELISA) plates coated with the corresponding synthetic peptides, and the specificity of hybridoma candidates for HLA-G isoforms was confirmed by Western blotting with HLA-G1–HLA-G6 standard proteins. Specific hybridomas were established by limiting-dilution cloning.

Isotyping of mAbs was performed using the Thermo Fisher Mouse Antibody Subtype Rapid Identification Kit (Pierce Rapid Isotyping Kit–Mouse; Cat. No. 26178). The antibody affinity constant was measured using ELISA. The recognition epitope of each mAb was analyzed using peptide ELISA with a sequence panel of mapping peptides based on the immunizing sequence. Optical density (OD) was measured at 450 nm (Multiskan FC; Thermo Scientific, Shanghai, China). ELISA was performed in quadruplicate, and results are expressed as mean OD ± SD.

### Study patients

A total of 345 consecutive patients with primary colorectal cancer (CRC) who underwent surgical treatment at Taizhou Hospital of Zhejiang Province between 21 May 2007 and 6 September 2017 were retrospectively included in this study. Patients with histopathologically confirmed CRC were eligible. None of the patients received preoperative radiotherapy, chemotherapy, or other medical interventions. Tumor samples were obtained from primary CRC lesions only; no specimens from metastatic sites were included. All samples underwent microscopic confirmation of pathological features before inclusion.

Formalin-fixed paraffin-embedded (FFPE) CRC samples, together with each patient’s medical history, clinicopathological data, and follow-up information, were retrieved from the Biological Resource Center, National Human Genetic Resources Platform of China (YCZYPT 2017), Taizhou Hospital of Zhejiang Province. CRC stage was determined according to the 7^th^ edition of the American Joint Committee on Cancer (AJCC) Cancer Staging System (33).

Of the 345 patients with CRC (*n* = 176 colon; *n* = 169 rectal), 204 were male and 141 were female (median age = 66 years). There were 70, 110, 159, and 6 patients with stage I, II, III, and IV disease, respectively. The latest follow-up for CRC-related events was completed on 20 January 2024, with a median follow-up period of 87.88 months (range, 1.37–204.33 months). Overall survival (OS) was calculated from the date of surgery to the date of the event or the most recent follow-up.

### Immunohistochemical analysis of HLA-G2/6 and HLA-G1/4/5 isoforms

Immunohistochemical (IHC) analysis of HLA-G2/6 and HLA-G1/4/5 isoforms in 345 case-matched CRC lesions was performed using mAb anti-HLA-G2/6 (clone YWHG-26) and mAb anti-HLA-G1/4/5 (clone YWHG-4). IHC staining of 4 µm FFPE CRC sections was conducted according to a standard protocol described previously (31). Briefly, slides were deparaffinized in xylene and rehydrated through a graded ethanol series. Endogenous peroxidase activity was blocked with 3% hydrogen peroxide, and antigen retrieval was performed at 120 °C in 10 mM sodium citrate buffer (pH 6.0). After blocking with 1% bovine serum albumin (BSA), CRC sections were incubated with anti-HLA-G2/6 mAb (1:500, clone YWHG-26) or anti-HLA-G1/4/5 mAb (1:500, clone YWHG-4) at 37 °C for 2 h. After washing, the sections were incubated with horseradish peroxidase (HRP)-conjugated rabbit/mouse secondary antibody (1:100; Dako, Glostrup, Denmark) at 37 °C for 30 min. IHC staining was developed using the Dako EnVision kit (Dako, Glostrup, Denmark), and counterstaining was performed with hematoxylin. Images of HLA-G2/6 and HLA-G1/4/5 staining were captured using 3DHistech (Budapest, Hungary).

The percentage of HLA-G2/6- and HLA-G1/4/5-positive tumor cells was independently evaluated by two reviewers blinded to patient information. The percentage of positive tumor cells was calculated based on staining positivity regardless of intensity. Tumor-cell membrane and/or cytoplasmic staining for HLA-G2/6 or HLA-G1/4/5 was considered positive. The percentage determined by each observer was averaged to obtain a final score. CRC samples with ≥ 5% tumor cells expressing HLA-G2/6 or HLA-G1/4/5 were considered positive, according to Chew et al. (34) (Tissue Antigens, 2007) [34], which defined complete loss of HLA class I expression as < 5% stained tumor cells per section.

Also, to analyze whether staining intensity affected the prognostic value of HLA-G2/6 and HLA-G1/4/5 expression in CRC, the immunoreactivity score (IRS) method was used (35) to further evaluate the significance of HLA-G2/6 and HLA-G1/4/5 expression for the survival of the CRC. In this study, the IRS was calculated as the product of the percentage of positive tumor cells (0, 0%; < 10%, 1; 10%–50%, 2; 51%–80%, 3; > 80%, 4) and staining intensity (0, none; 2, moderate; 3, strong), yielding IRS values between 0 (no staining) and 12 (maximum staining).

### Statistical analysis

The associations between HLA-G2/6 and HLA-G1/4/5 expression and clinicopathological variables—including sex, age, tumor site (colon or rectum), TNM classification, and AJCC stage—were analyzed using the chi-square test. Patient survival was analyzed using the Kaplan–Meier method with the log-rank test. The prognostic significance of variables, including HLA-G2/6 and HLA-G1/4/5 isoform expression status, was assessed using the univariate Cox proportional hazards model. Variables with a *p*-value ≤ 0.20 in univariate analysis were included in the multivariate Cox regression model.

All statistical analyses and plotting were performed using SPSS (version 13.0; SPSS Inc., Chicago, IL, USA). Statistical significance was set at *p* < 0.05. Hazard ratios (HRs) and 95% confidence intervals (CIs) were calculated to estimate the relative risk.

## Results

### HLA-G1~HLA-G6 isoform cloning and expression

Total RNA was extracted from HLA-G–positive JEG-3 cells, and transcripts for the HLA-G1–HLA-G6 isoforms were successfully obtained. PCR products of the expected sizes corresponding to each isoform were ligated into the pGEM-T vector and verified by PCR and sequencing. Sequence alignment revealed that the cDNA sequence of the HLA-G isoforms was identical to that of the transcript HLA-G*010103 (data not shown).

Western blot analysis of HLA-G1–HLA-G6 expressed in K562 cells was performed using mAb 4H84, while HLA-G5 and HLA-G6 were additionally verified with mAb 5A6G7. The expected molecular weights of the HLA-G1, HLA-G2, HLA-G3, HLA-G4, HLA-G5, and HLA-G6 isoforms were approximately 39, 31, 23, 30, 37, and 27 kDa, respectively (**Supplementary Figure 2A**). Expression of HLA-G5 and HLA-G6 was further confirmed with mAb 5A6G7 (**Supplementary Figure 2B**).

### Characteristics of the anti-HLA-G2/6 and anti-HLA-G1/4/5 antibodies

The purity of the anti–HLA-G2/6 and anti–HLA-G1/4/5 mAbs was confirmed by sodium dodecyl sulfate–polyacrylamide gel electrophoresis (SDS–PAGE) (**Supplementary Figures 3A, B**). The anti–HLA-G2/6 antibody was classified as IgG2a (κ), and anti–HLA-G1/4/5 as IgG1 (κ) (**Supplementary Figures 3C, D**). The affinity constants (K) of anti–HLA-G2/6 and anti–HLA-G1/4/5 were 1.42 × 10^9^ L/mol and 9.87 × 10_9_ L/mol, respectively (**Supplementary Figures 3E, F**).

**Figure 1:**
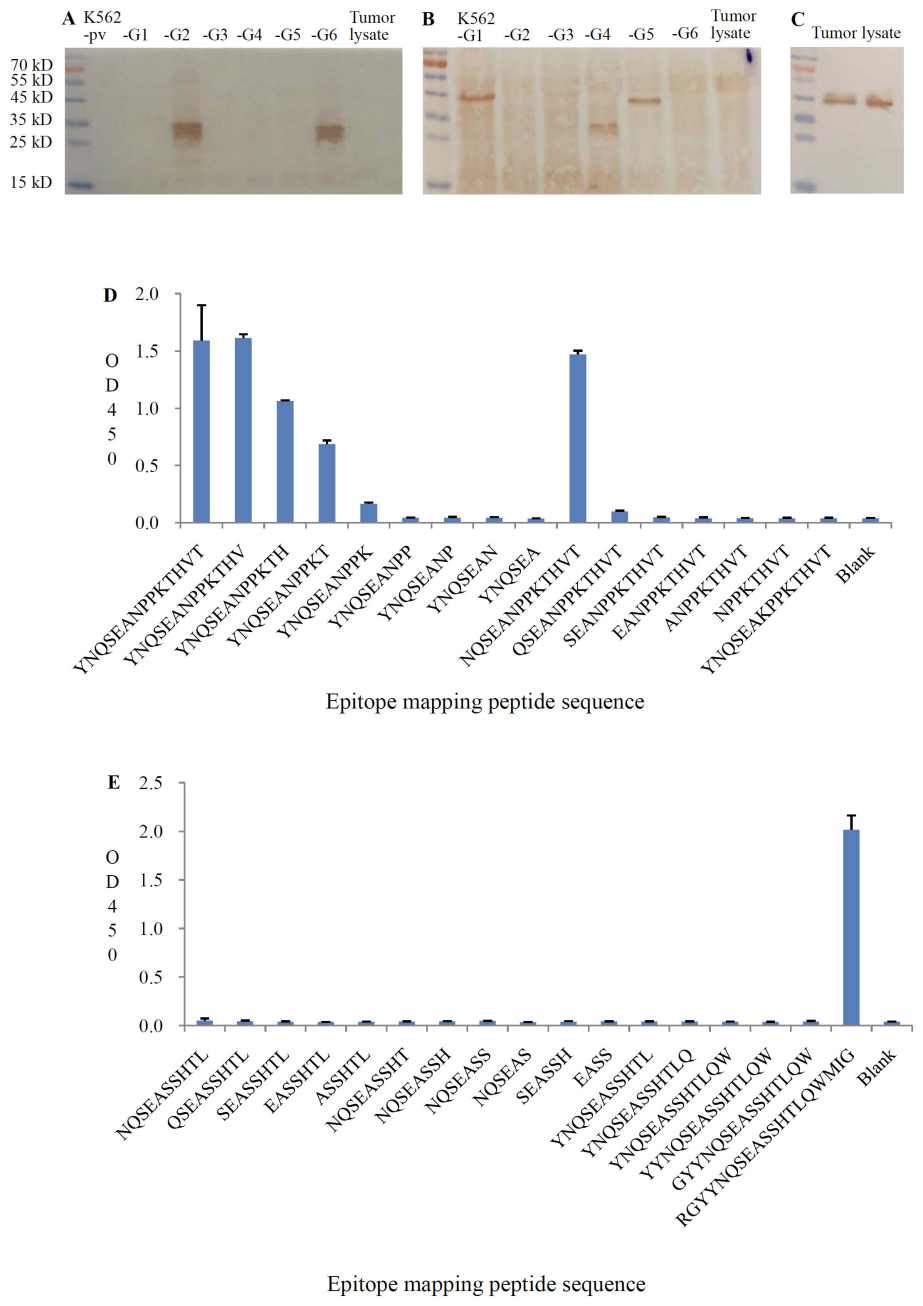
Specificity of anti-HLA-G2/6 and anti-HLA-G1/4/5 analyzed with western blot analysis. **(A)** HLA-G1 to HLA-G6/K562 tranfectant cell lysates and tumor lysates were probed with anti-HLA-G2/6 (1:1000). **(B)** HLA-G1 to HLA-G6/K562 tranfectant cell lysates and tumor lysates were probed with anti-HLA-G1/4/5 (1:1000). **(C)** Tumor lysates were probed with anti-HLA-A, HLA-B, HLA-C, and HLA-E (1:1000, clone TP25.99SF, Exbio). Recognition epitopes of the mAbs anti-HLA-G2/6 **(D)** and anti-HLA-G1/4/5 **(E)** were analyzed with a specific sequence panel of the mapping peptide based on the immunized peptide with peptide ELISA.

The specificity of these antibodies was confirmed by Western blotting using lysates from K562 cells expressing HLA-G1–HLA-G6 isoforms. Anti–HLA-G2/6 was specific for the HLA-G2 and HLA-G6 isoforms but did not cross-react with other HLA class I antigens (**Figure 1A**). Similarly, anti–HLA-G1/4/5 was specific for the HLA-G1, HLA-G4, and HLA-G5 isoforms (**Figure 1B**).

No cross-reactivity was detected with other HLA-G isoforms or classical HLA class I molecules (HLA-A, -B, -C, and -E), as confirmed using the mAb TP25.99SF (Exbio, Czech Republic) (**Figure 1C**).

Epitope mapping showed that the anti–HLA-G2/6 mAb recognized the specific peptide sequence YNQSEAKPPKT, located in the junction region between the α1 and α3 domains shared by HLA-G2 and HLA-G6 (**Figure 1D**). The anti–HLA-G1/4/5 mAb recognized the specific peptide sequence RGYYNQSEASSHTLQWMIG, located in the junction region between the α1 and α2 domains shared by HLA-G1, HLA-G4, and HLA-G5 (**Figure 1E**).

### Relevance of the HLA-G2/6 and HLA-G1/4/5 isoforms in patients with CRC

**Figure 2:**
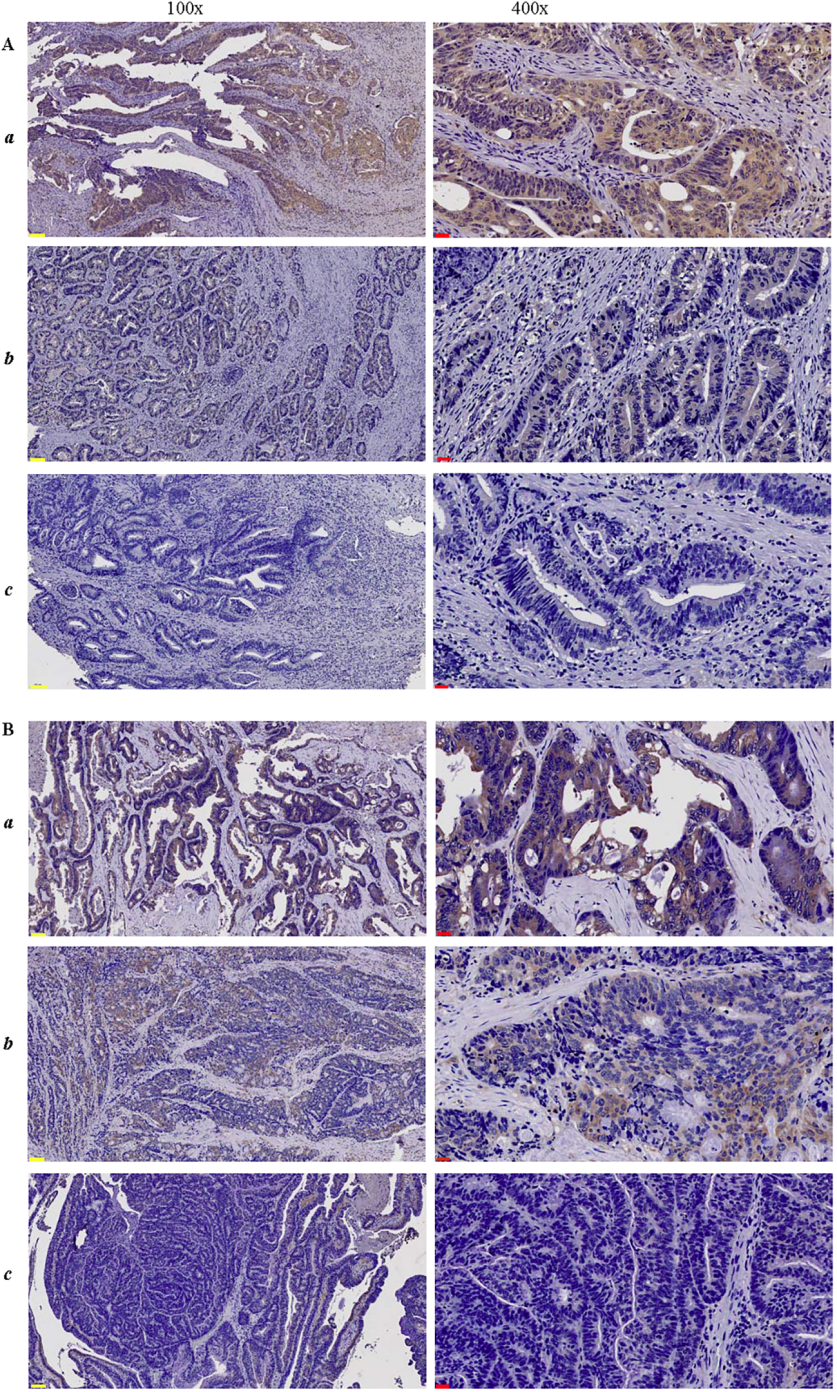
Immunohistochemical staining of CRC lesions with **(A)** anti-HLA-G2/6 (1:500) and **(B)** anti-HLA-G1/4/5 (1:500) at 100× and 400× magnification, respectively. (a-c) indicate strongly positive, weak or moderate positive, and negative staining. Scale bars (yellow) measure 100μm, and (black) measure 20μm.

The immunohistochemical (IHC) staining results are shown in **Figure 2**. IHC analysis revealed that HLA-G2/6 and HLA-G1/4/5 expression was heterogeneous among the case-matched CRC samples. A significant difference in expression rates was observed between HLA-G2/6 (26.1%, 90/345) and HLA-G1/4/5 (79.7%, 275/345; *p* < 0.001; **Table 1**).

**Table 1:** HLA-G2/6 and HLA-G1/4/5 expression related to the clinical parameters in CRC patients.

Variables	Cases	HLA-G2/6		*p*	HLA-G1/4/5		*p*
		Neg.	Pos. (%)		Neg.	Pos. (%)	
Total	345	255	90 (26.1%)		70	275 (79.7%)	<0.001
Sex							
Male	204	154	50 (24.5%)	0.422	36	168 (82.4%)	0.142
Female	141	101	40 (28.4%)		34	107 (75.9%)	
Age							
≤ median (66 ys)	174	127	47 (27.0%)	0.693	36	138 (79.3%)	0.852
> median (66 ys)	171	128	43 (25.1%)	34	137 (80.1%)		
Type							
Colon	176	128	48 (27.3%)	0.609	38	138 (78.4%)	0.540
Rectal	169	127	42 (24.9%)		32	137 (81.1%)	
TNM stage							
pT							
T1 + 2	102	75	27 (26.5%)	0.871	16	86 (84.3%)	0.271
T3	234	174	60 (25.6%)		51	183 (78.2%)	
T4	9	6	3 (33.3%)	3	6 (66.7%)
pN							
N0	182	134	48 (26.4%)	0.977	39	143 (78.6%)	0.857
N1	95	71	24 (25.3%)		18	77 (81.1%)	
N2	68	50	18 (26.5%)	13	55 (80.9%)
pM							
M0	339	253	86 (25.4%)	0.022	69	270 (79.6%)	0.824
M1	6	2	4 (66.70%)		1	5 (83.3%)	
AJCC stage							
I	70	49	21 (30.0%)	0.410	14	56 (80.0%)	0.884
II	110	84	26 (23.6%)		25	85 (77.3%)	
III	159	120	39 (24.5%)	30	129 (81.1%)
IV	6	2	4 (66.70%)	1	5 (83.3%)

^*^ Comparison of HLA-G2/6 and HLA-G1/4/5 expression between or among each variable using the Pearson chi-square test.

Statistical analysis showed that neither HLA-G2/6 nor HLA-G1/4/5 expression was significantly associated with demographic or clinical characteristics of patients with CRC, such as sex, age, tumor location, pT, pN, or AJCC stage. However, a markedly higher proportion of HLA-G2/6 expression was observed in patients with pM1 status (*p* = 0.022; **Table 1**). It should be noted that this cohort included only six patients with pM1 disease; thus, the statistical significance of the higher HLA-G2/6 expression in this subgroup may be limited.

Regarding HLA-G2/6 and HLA-G1/4/5 co-expression (distribution shown in **Supplementary Figure 4**), the 345 case-matched CRC samples comprised the following groups: HLA-G2/6^+^ HLA-G1/4/5^+^ (n = 79), HLA-G2/6^+^ HLA-G1/4/5^−^ (n = 11), HLA-G2/6^−^ HLA-G1/4/5^+^ (n = 196), and HLA-G2/6^−^ HLA-G1/4/5^−^ (n = 59). Co-expression status was not significantly associated with any demographic or clinical characteristics of patients with CRC (**Supplementary Table 2**).

### Prognostic value of HLA-G2/6 and HLA-G1/4/5 expression in patients with CRC

**Figure 3:**
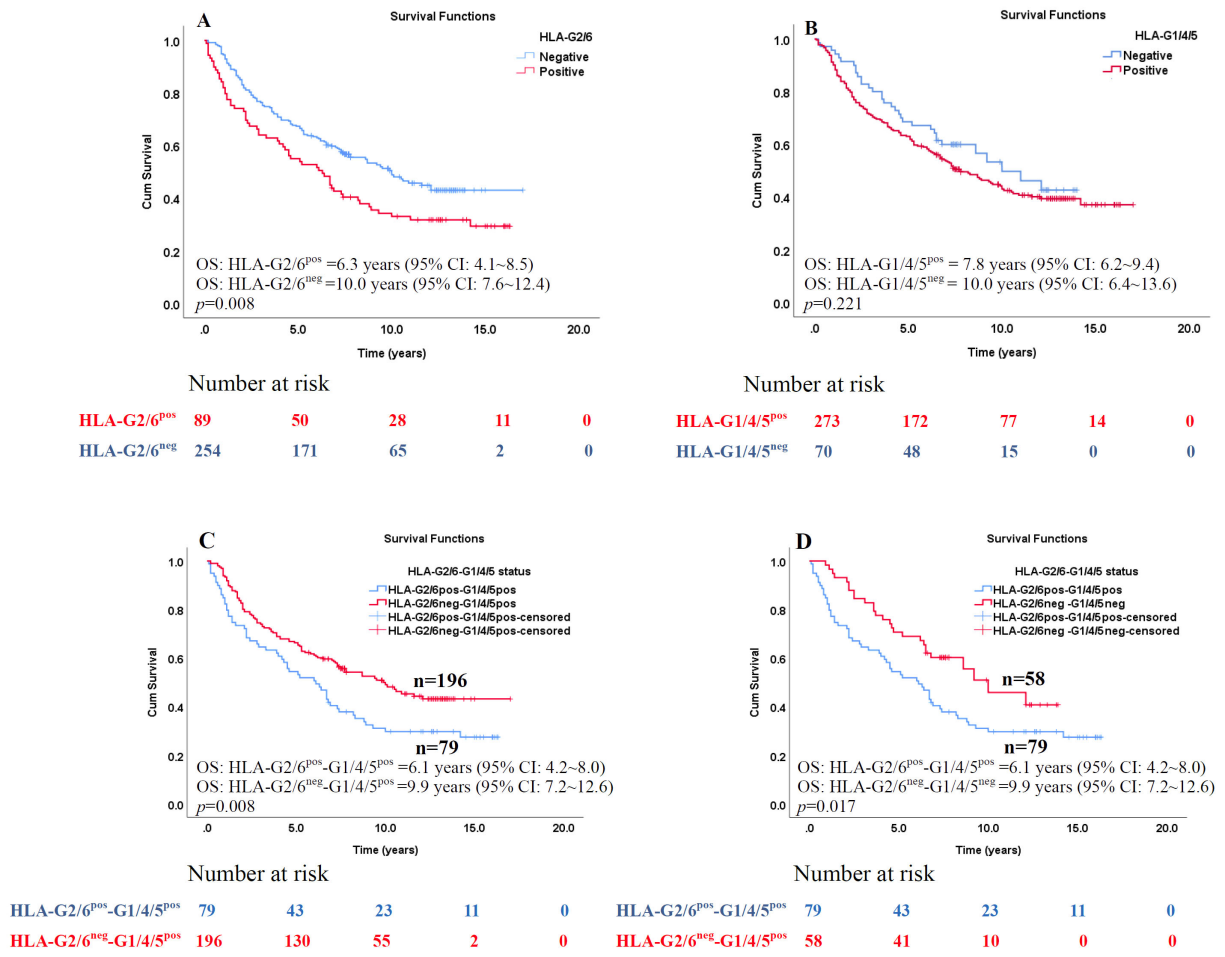
Kaplan–Meier survival analysis of the percentages of HLA-G2/6 and HLA-G1/4/5 isoform expression in patients with CRC. Comparison of overall survival between CRC patients with **(A)** HLA-G2/6 isoform negative- and positive- expression (*p* = 0.008), **(B)** HLA-G1/4/5 isoform negative- and positive-expression (*p* = 0.221), **(C)** Co-expression of HLA-G2/6^pos^-G1/4/5^pos^ and HLA-G2/6^neg^-G1/4/5^pos^ (*p* = 0.008), and **(D)** Co-expression of HLA-G2/6^pos^-G1/4/5^pos^ and HLA-G2/6^neg^ -G1/4/5^neg^ (*p* = 0.017).

The prognostic significance of HLA-G2/6 and HLA-G1/4/5 expression was assessed using log-rank (Mantel–Cox) analysis (**Supplementary Table 3**). Patients with HLA-G2/6 expression had significantly poorer prognosis than those without expression [median OS, 6.3 years (95% CI, 4.1–8.5) vs. 10.0 years (95% CI, 7.6–12.4); *p* = 0.008; **Figure 3A**], whereas HLA-G1/4/5 expression was not significantly associated with survival [median OS, 10.0 years (95% CI, 6.4–13.6) *vs.* 7.8 years (95% CI, 6.2–9.4); *p* = 0.221; **Figure 3B**].

Female patients (*p* = 0.024), younger patients (*p* = 0.010), and those with earlier pN (*p* < 0.001) or AJCC stage (*p* = 0.004) exhibited significantly better survival (**Supplementary Table 3**).

Among co-expression subgroups (HLA-G2/6^pos^HLA-G1/4/5^pos^, HLA-G2/6^pos^HLA-G1/4/5^neg^, HLA-G2/6^neg^HLA-G1/4/5^pos^ and HLA-G2/6^neg^ HLA-G1/4/5^neg^ groups, patients with HLA-G2/6^pos^ HLA-G1/4/5^pos^ showed significantly shorter survival than those with HLA-G2/6^neg^HLA-G1/4/5^pos^ [median OS: 6.1 years (95% CI: 4.2~8.0) *vs.* 9.9 years (95%CI: 7.2~12.6), *p* = 0.008;**Figure 3C**] and HLA-G2/6^neg^HLA-G1/4/5^neg^ [median OS: 6.1 years (95%CI: 4.2~8.0) *vs.* 9.9 years (95%CI: 7.2~12.6), *p* = 0.017; **Figure 3D**]. No other pairwise comparisons were statistically significant (data not shown).

To further evaluate the prognostic value, we used the immunoreactivity score (IRS) method. Based on IRS, HLA-G2/6 and HLA-G1/4/5 expression were categorized as negative (IRS = 0), moderate [IRS≤median of the positive group (IRS:1~12)] and strong [IRS>median of the positive group (IRS:1~12)]. The median IRS was 4.0 (range, 1.0–12) for HLA-G2/6-positive samples and 6.0 (range, 1.0–12) for the HLA-G1/4/5-positive group, respectively. Details of the IRS distribution and groups (negative, moderate positive and strong positive) of the HLA-G2/6 and HLA-G1/4/5 expression according to IRS are shown in **Supplementary Figure 5**.

**Figure 4:**
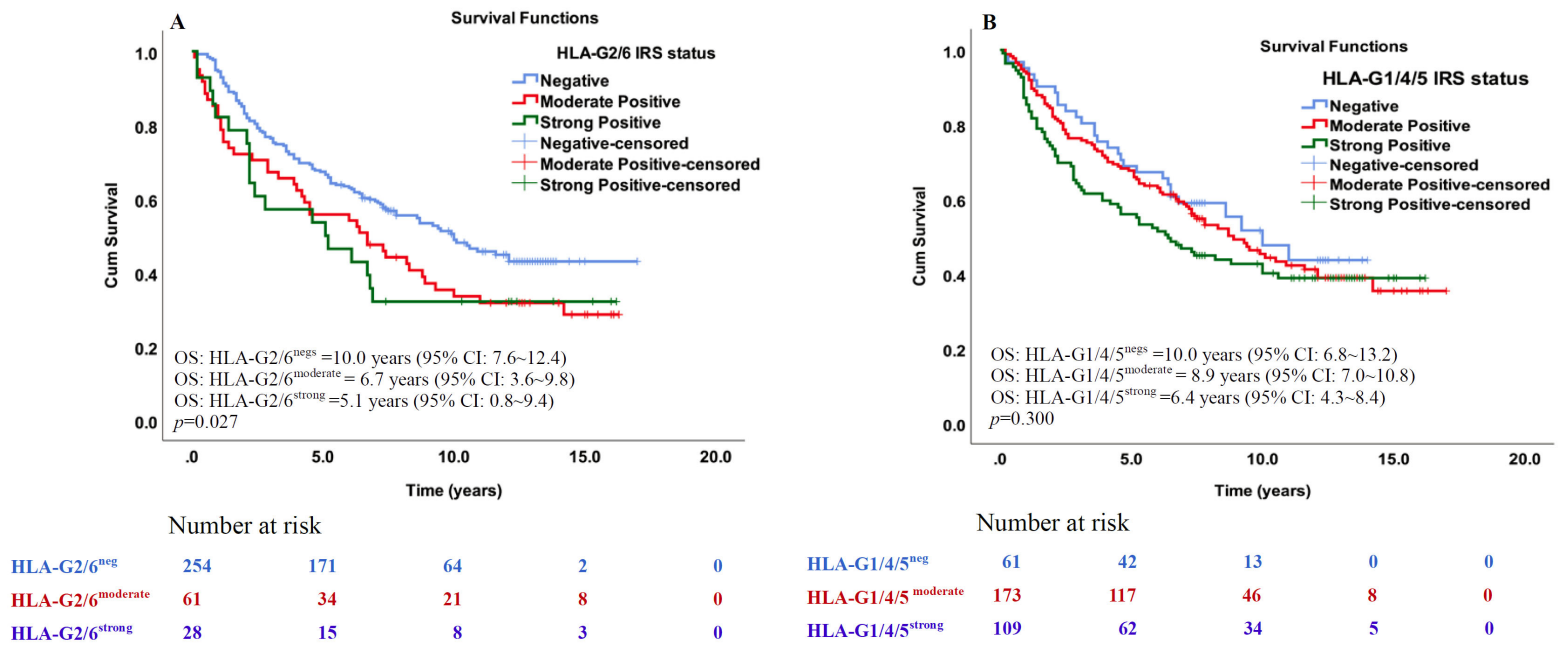
Kaplan–Meier survival analysis of the IRS degree of HLA-G2/6 and HLA-G1/4/5 expression in patients with CRC. Comparison of overall survival between CRC patients with **(A)** HLA-G2/6 isoform negative-, moderate- and strong expression (*p* = 0.027), **(B)** HLA-G1/4/5 isoform negative-, moderate- and strong expression (*p* = 0.300).

Log-rank Mantel-Cox analysis showed that IRS status of HLA-G2/6 expression was significantly associated with poorer survival [median OS^HLA-G2/6neg^: 10.0 years (95% CI: 7.6 ~12.4; n=254); OS^HLA-G2/6moderate^: 6.7 years (95% CI: 3.6 ~9.8; n=61); and OS^HLA-G2/6strong^: 5.1 years (95% CI: 0.8 ~9.4; n=28); *p* = 0.027, **Figure 4A**], whereas IRS status of HLA-G1/4/5 expression was not statistically significant for the prognosis of CRC [median OS^HLA-G1/4/5neg^: 10.0 years (95% CI: 6.8 ~13.2; n=61); OS ^HLA-G1/4/5moderate^: 8.9 years (95% CI: 7.0 ~10.8; n=173); and OS ^HLA-G1/4/5strong^: 6.4 years (95% CI: 4.3 ~8.4; n=109); *p* = 0.300, **Figure 4B**]. Regarding the two scoring methods—one using only the percentage of positive cells stained and the other incorporating staining intensity—both revealed that patients with HLA-G2/6, but not HLAG1/4/5, expression were significantly associated with poorer overall survival of CRC.

We then entered the significant factors related to CRC patient survival, including sex, age, AJCC stage, and percentage status of HLA-G2/6 expression (which was related to CRC patient survival) into a univariate and subsequent multivariate Cox proportional hazard model analysis. The hazard ratios (HRs) for female sex, older patient age, advanced AJCC stage, and positive HLA-G2/6 expression were 0.735 (95% CI: 0.542~0.995, *p* = 0.046), 1.467 (95% CI: 1.097~1.962, *p* = 0.010), 1.747 (95% CI: 1.307~2.335, *p* < 0.001), and 1.530 (95% CI: 1.125~2.081, *p* = 0.007), respectively. These results identified HLA-G2/6 expression as an independent prognostic factor for poor survival in patients with CRC (**Table 2**).

**Table 2:** Cox proportional hazards model analysis of variables affecting overall survival in CRC patients.

Variables	Categories	Univariate analysis		Multivariate analysis	
		HR (95% CI)	*P*	HR (95% CI)	*P*
Sex	Female vs male	0.708 (0.524~0.958)	0.025	0.735 (0.542~0.995)	0.046
Age (years)	>66 *vs ≤*66	1.457 (1.092~1.945)	0.011	1.467 (1.097~1.962)	0.010
AJCC stage	III/IV *vs* I/II	1.653 (1.239~2.204)	0.001	1.747 (1.307~2.335)	<0.001
HLA-G2/6	Pos *vs* Neg	1.512 (1.112~2.055)	0.008	1.530 (1.125~2.081)	0.007

HR=hazard ratio; 95% CI = 95% confidence interval.

### Prognostic value of HLA-G2/6 expression in stratified patients with CRC

The significance of prognostic stratification of biomarkers, including HLA-G, has been observed in various cancers (36). Therefore, we evaluated the prognostic stratification value of HLA-G2/6 expression in subgroups of patients with CRC. Positive HLA-G2/6 expression was associated with worse survival among female patients (7.6 years *vs.* 10.4 years, *p* = 0.003), younger patients (7.1 years *vs.* 10.5 years, *p* < 0.001), those with colon cancer (7.8 years *vs.* 10.5 years, *p* = 0.045), and patients with pT3 (*p* = 0.008), pN1 (*p* = 0.020), pM0 (*p* = 0.009), and AJCC III (*p* = 0.005) disease (**Table 3**). In contrast, HLA-G1/4/5 expression showed no prognostic significance in any subgroup (data not shown).

**Table 3:** Log-rank Mantel–Cox analysis of the prognostic stratification significance HLA-G2/6 for CRC patients.

		HLA-G2/6 negative		HLA-G2/6 positive		
Variables	Stratified variables	No. event/total	Survival (year) (95% CI)	No. event/total	Survival (year) (95% CI)	*P*
Sex	Male	88/153	9.1 (8.1~10.2)	36/50	7.8 (6.1~9.4)	0.274
	Female	39/101	10.4 (9.3~11.5)	25/39	7.6 (5.7~9.7)	0.003
Age	≤66 ys	50/126	10.5 (9.5~11.5)	33/46	7.1 (5.4~8.9)	<0.000
	>66ys	77/128	8.5 (7.3~9.6)	28/43	8.2 (6.3~10.1)	0.951
Type	Colon	60/128	10.5 (9.3~11.7)	31/47	7.8 (5.9~9.6)	0.045
	Rectal	67/126	8.8 (7.8~9.8)	30/42	7.6 (5.8~9.4)	0.115
pT	T_1 + 2_	39/75	10.4 (8.9~11.9)	15/27	8.7 (6.1~11.3)	0.517
	T_3_	86/173	8.6 (7.8~9.4)	45/60	7.1 (5.6~8.61)	0.008
	T_4_	2/6	9.7 (5.2~14.2)	1/2	9.3 (0.0~19.0)	0.867
pN	N_0_	59/133	11.0 (9.8~12.1)	29/48	9.3 (7.5~11.0)	0.235
	N_1_	36/71	9.3 (7.9~11.0)	18/23	6.4 (4.1~8.7)	0.020
	N_2_	32/50	6.7 (5.2~8.2)	14/18	4.4 (2.2~6.6)	0.148
pM	M_0_	126/252	10.1 (9.2~10.9)	59/86	7.7 (6.3~9.0)	0.009
	M_1_	1/2	7.5 (0.0~16.1)	2/3	5.8 (1.3~10.4)	0.984
AJCC	I	19/49	12.2 (10.5~13.9)	11/21	9.0 (6.0~12.0)	0.211
	II	39/83	9.0 (7.9~10.1)	17/26	9.5 (7.4~11.6)	0.769
	III	68/120	8.3 (7.2~9.4)	31/39	5.7 (3.9~7.5)	0.005
	IV	1/2	7.5 (0.0~16.1)	2/3	5.8 (1.3~10.4)	0.984

## Discussion

In addition to the well-acknowledged roles of classical HLA class I antigens in tumor recognition and immune surveillance, non-classical HLA class I molecules (HLA-E, HLA-F, HLA-G, and HLA-H) have gained attention for their roles in shaping the tumor microenvironment (TME) and influencing immune responses. Further understanding of the clinical relevance of non-classical HLA class I molecules could offer new insights into cancer immunology and lead to the development of innovative and more effective immunotherapeutic approaches (6, 37, 38). In this regard, challenges such as the biological roles and clinical significance of specific individual HLA-G isoforms and their co-expression with other HLA-G isoforms, as well as the balance of HLA-E receptors (the inhibitory receptor CD94/NKG2A and activating receptor CD94/NKG2C) in the TME, remain to be further explored (7, 11, 39).

In this study, for the first time, we successfully developed and characterized anti-HLA-G2/6 and anti-HLA-G1/4/5 monoclonal antibodies. These two new antibodies against HLA-G isoforms are important for advancing the exploration and understanding of HLA-G biology and its clinical implications. Our findings revealed that HLA-G2/6, but not HLA-G1/4/5, was significantly associated with poor survival and served as an independent prognostic indicator in CRC.

The novel immune checkpoint HLA-G has unique features, including lack of expression in normal tissue, pan-cancer-specific expression, potent immune suppression through signaling with the inhibitory receptors ILT-2 and ILT-4, and its association with poor prognosis in cancer patients, making HLA-G an attractive non-self and tumor-site-agnostic target for cancer immunotherapy. Indeed, clinical trials exploring various HLA-G-targeted immunotherapy strategies for diverse solid tumors have been initiated since 2020 (https://clinicaltrials.gov/search?cond=HLA-G). However, at least seven HLA-G isoforms (HLA-G1 to HLA-G7) with distinct molecular structures and receptor-binding characteristics are generated by alternative splicing. Because of the lack of isoform-specific mAbs, the clinical significance of most HLA-G isoforms, except HLA-G1 and HLA-G5, remains largely unknown (6). Furthermore, heterogeneity of HLA-G isoform expression in cancer lesions is frequently observed, raising major concerns about the precision of HLA-G-targeted cancer immunotherapy (11, 28). In the context of precision medicine, it is therefore critically important to develop HLA-G isoform-specific antibodies to explore the clinical relevance of distinct HLA-G isoform expression in cancer patients, which could facilitate more precise HLA-G-targeted immunotherapeutic strategies.

Since HLA-G expression was first observed in melanoma, over the last three decades, its expression has been explored in more than 30 types of pathological cancers, including CRC (8, 36, 40–42). Although studies have generally revealed that HLA-G expression varies dramatically among studies and even within the same type of cancer (11, 32, 43). These divergent observations could mainly be attributed to the fact that mAb 4H84-positive staining only indicates the total expression of all isoforms and cannot determine the individual profiles of the HLA-G1 to HLA-G7 isoforms or the degree of expression in cancer lesions for the detection of all α1 domain-retaining HLA-G isoforms (HLA-G1 to HLA-G7) (30). A panel of meta-analyses revealed that HLA-G expression detected using mAb 4H84 was significantly associated with worse survival, and that a much higher HR was observed in patients with CRC. However, remarkable heterogeneity in the HLA-G expression rate detected by mAb 4H84 (25% - 70%) and intensity has been found among patients with CRC (40, 44–46). Zhang et al. (45) reported that higher HLA-G expression is significantly associated with worse survival in patients with CRC and colon cancer, but not in those with rectal cancer. Consistent with these results, our finding showed that HLA-G2/6, but not HLA-G1/4/5 status, is significantly associated with the survival of patients with colon cancer (*p* = 0.045), but not in those with rectal cancer, revealing that distinct HLA-G isoforms could have unique clinical relevance.

Because of these limitations, the development of HLA-G isoform-specific antibodies capable of distinguishing individual isoforms and clarifying their clinical relevance is essential for improving our understanding of the biological roles of HLA-G isoforms and for enabling more precise HLA-G-targeted cancer immunotherapy (8, 27, 28). Current clinical trials (NCT04485013, NCT04991740, NCT06380816, NCT05672459, and NCT05769959) are based on the rationale of blocking HLA-G and its receptors ILT-2 and ILT-4, which bind the HLA-G1, HLA-G2, HLA-G5, and HLA-G6 isoforms. However, patients who are mAb 4H84^pos^ but lack the α3 domain—including those expressing HLA-G3, HLA-G4, and HLA-G7 isoforms—may not benefit from such immunotherapies In this regard, the first completed clinical trial (NCT04991740) involved 39 heavily pretreated patients with colorectal, ovarian, and renal cell carcinomas who received the CD3/HLA-G bispecific antibody JNJ-78306358 (21, 47). No objective responses were observed, and the trial was terminated because of progressive disease. In that study, 12 of the 25 lesions were HLA-G-positive as detected by mAb 4H84; however, the effect of HLA-G expression on treatment efficacy was not discussed, and thus the potential benefits of JNJ-78306358 therapy in patients with HLA-G expression remain unclear.

ILT-2 and ILT-4 are the major receptors for HLA-G and contain four and three immunoreceptor tyrosine-based inhibitory motifs (ITIMs) in their cytoplasmic tails, respectively. ILT-2 and ILT-4 are differentially expressed on various immune cells, including T cells, B cells, NK cells, DCs, neutrophils, invariant NKT cells, MDSCs, macrophages, and also on tumor cells (10, 48). Mechanistically, HLA-G/ILT-2 or HLA-G/ILT-4 signaling can comprehensively suppress both innate and adaptive anti-tumor immune responses by inhibiting the cytolytic functions of immune-competent cells and promoting immune-regulatory cell proliferation and accumulation, creating a profoundly immunosuppressive and pro-neoplastic TME (28, 29). ILT-2 and ILT-4 recognize the extracellular α3 domain of HLA-G, although their binding specificities differ markedly. ILT-2 binding is β_2_m-dependent and interacts only with β_2_m-associated isoforms (HLA-G1/β_2_m and HLA-G5/β_2_m). In contrast, ILT-4 binding is β_2_m-independent and can recognize both β_2_m-free and β_2_m-associated HLA-G isoforms, including HLA-G1, HLA-G2, HLA-G5, and HLA-G6 (49, 50). Notably, receptor(s) for the HLA-G3 and HLA-G4 isoforms remain unknown yet (51). In addition to immune cells, tumor cells themselves also express ILT-2 and ILT-4. Previous studies have shown that ILT-4 expression in cancer can enhance vascular endothelial growth factor (VEGF)-C expression, promoting tumor metastasis and disease relapse (52–54). Moreover, only ILT-4 expression in tumor cells has been significantly associated with poor survival in patients with CRC and gastric cancer (36, 55).

With the anti-HLA-G2/6 and anti-HLA-G1/4/5 specific monoclonal antibodies, our study is the first to demonstrate the heterogeneity of HLA-G2/6 and HLA-G1/4/5 isoform expression in patients with CRC, and that the expression rate of HLA-G2/6 (26.1%) was remarkably lower than that of HLA-G1/4/5 (79.7%). More importantly, only HLA-G2/6 expression was significantly associated with poorer survival and was an independent prognostic indicator. Furthermore, HLA-G2/6 showed significance for prognostic stratification among subgroups of patients with CRC, indicating that HLA-G2/6 could significantly affect survival among female patients, younger patients, patients with colon cancer, and patients at pT3, pN1, pM0, or AJCC III stage. In addition, patients with HLA-G2/6^+^-HLA-G1/4/5^+^ tumors had significantly shorter survival than those with HLA-G2/6^neg^-G1/4/5^pos^ and HLA-G2/6^neg^- HLA-G1/4/5^neg^, further indicating that HLA-G2/6, rather than HLA-G1/4/5, might be the risk factor for worse survival in CRC. However, our findings on the stratified prognostic value of HLA-G2/6 in CRC subgroups are preliminary, and the detailed significance of HLA-G2/6 or HLA-G1/4/5 expression—or their co-expression—requires further investigation, such as through studies using mouse models.

Based on our findings, disease progression and poor prognosis in patients with CRC might result from the different binding specificities and affinities between ILT-2/ILT-4 and their ligand HLA-G isoforms (50). Moreover, multimerization of HLA-G isoforms affects ILT-2/ILT-4 binding affinity. In line with this, a study by Kuroki et al. (56) revealed that HLA-G2, and possibly its soluble form HLA-G6, features an HLA class II-like heterodimer, and that the HLA-G2 homodimer binds to ILT-4 with slower dissociation and significantly higher avidity than the HLA-G1 homodimer. These studies suggest that HLA-G2/6 isoforms—homodimers in particular—may exert stronger immunosuppressive effects than other HLA-G isoforms, thereby promoting cancer progression. However, the precise underlying mechanisms involved in HLA-G2/6 and HLA-G1/4/5 expression and their clinical implications in CRC remain to be explored. An increasing number of studies have highlighted that key cancer-associated neoantigens and spliceosomal proteins are frequently altered in cancer, leading to pathogenesis and/or treatment resistance (57), such as in the HER2 and BRCA1 isoforms (58, 59). However, the alternative splicing mechanisms regulating HLA-G isoform expression are poorly studied. To the best of our knowledge, a study by Leisegang et al. (60) revealed that the histone demethylase plant homeodomain finger protein 8 (PHF8) specifically interacts with U1-70K and SRPK1 (components of the U1 snRNP splicing machinery), and that PHF8 is important for HLA-G intron 4 exclusion through regulation of local H3K9me2. As a result, depletion of PHF8 generates only soluble HLA-G isoforms rather than membrane-bound isoforms. In this regard, in the context of precision medicine, our study highlights the need to explore the alternative splicing mechanisms involved in the regulation of HLA-G isoform expression and to perform HLA-G isoform typing for HLA-G-targeted cancer immunotherapy.

Obviously, our study has limitations. First, it was based on a single-center, retrospective design with a limited number of patients with CRC. The real-world expression of HLA-G2/6 and HLA-G1/4/5 isoforms in other cancer types, and in multi-center, larger cohorts, remains to be investigated. Second, the clinical relevance of other HLA-G isoforms not detected by anti-HLA-G2/6 and anti-HLA-G1/4/5 mAbs cannot be excluded. Third, the mechanisms underlying the differential expression of HLA-G2/6 and HLA-G1/4/5 isoforms in CRC are yet to be uncovered.

In conclusion, this is the first study to generate mAbs for the HLA-G2/6 and HLA-G1/4/5 isoforms. The findings revealed that HLA-G2/6, but not HLA-G1/4/5, expression is an independent prognostic indicator for poor survival in patients with CRC. Moreover, combining HLA-G2/6 expression with demographic or clinical characteristics could further improve prognostic assessment for particular CRC subgroups. Our findings are of great importance for clarifying the clinical relevance of HLA-G2/6 and HLA-G1/4/5 expression in other cancers and for advancing precision HLA-G-targeted cancer immunotherapy for patients with solid tumors.

## Data Availability

The original contributions presented in the study are included in the article/**Supplementary Material**. Further inquiries can be directed to the corresponding author.

## Supplementary material

Supplementary Figure 1 Generation of anti-HLA-G2/6 and anti-HLA-G1/4/5 antibodies. **(A)** anti-HLA-G2/6 generated by an immunogen located in the junction region between α1 and α3 (RGYYNQSEAKPPKTHVTHHPV) specifically shared by HLA-G2 and HLA-G6 (indicated by dashed frame). **(B)** Anti-HLA-G1/4/5 generated by an immunogen peptide located in the junction region between α1 and α2 (RGYYNQSEASSHTLQWMIG) specifically shared by HLA-G1, HLA-G4, and HLA-G5 (indicated by dashed frame).

Supplementary Figure 2 Generation of HLA-G1–HLA-G6 isoforms was confirmed by western blotting. **(A)** HLA-G1–HLA-G6/K562 transfected cell lysates were probed with mAb 4H84 (1:1000, Exbio). **(B)** HLA-G1–HLA-G6/K562 transfected cell lysates were probed with mAb 5A6G7 (1:1000, Exbio).

Supplementary Figure 3 Characteristics of the anti-HLA-G2/6 and anti-HLA-G1/4/5 antibodies. SDS-PAGE purity analysis of **(A)** anti-HLA-G2/6 and **(B)** anti-HLA-G1/4/5; isotyping of **(C)** anti-HLA-G2/6 and **(D)** anti-HLA-G1/4/5; affinity constant curves of **(E)** anti-HLA-G2/6 and **(F)** anti-HLA-G1/4/5.

Supplementary Figure 4 Distribution of the co-expression of HLA-G2/6 and HLA-G1/4/5 in patients with CRC.

Supplementary Figure 5 Distribution of IRS and grouping (negative, moderate, and strong) of HLA-G2/6 and HLA-G1/4/5 expression in patients with CRC.
