# Supplementary figures and images for: Human leukocyte antigen-G isoform HLA-G2/6, but not HLA-G1/4/5, is an independent indicator of poor survival in patients with colorectal cancer
Source: Front Immunol. 2025 Oct 21;16:1672144. doi: 10.3389/fimmu.2025.1672144 (PMC12583952; doi:10.3389/fimmu.2025.1672144)

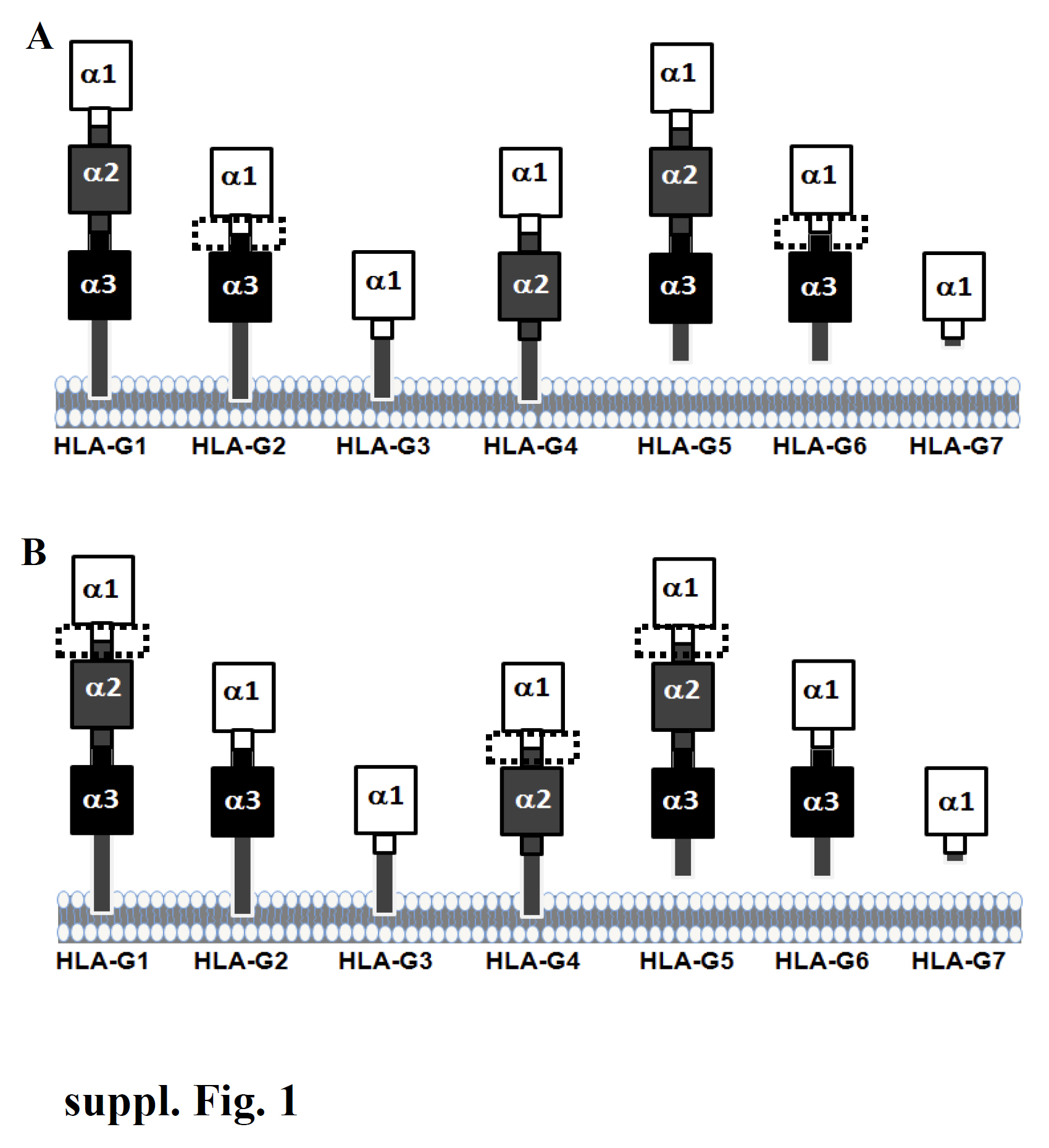

Supplement: Supplementary Figure 1 — Generation of anti-HLA-G2/6 and anti-HLA-G1/4/5 antibodies. (A) anti-HLA-G2/6 generated by an immunogen located in the junction region between α1 and α3 (RGYYNQSEAKPPKTHVTHHPV) specifically shared by HLA-G2 and HLA-G6 (indicated by dashed frame). (B) Anti-HLA-G1/4/5 generated by an immunogen peptide located in the junction region between α1 and α2 (RGYYNQSEASSHTLQWMIG) specifically shared by HLA-G1, HLA-G4, and HLA-G5 (indicated by dashed frame). [file Image1.jpeg]

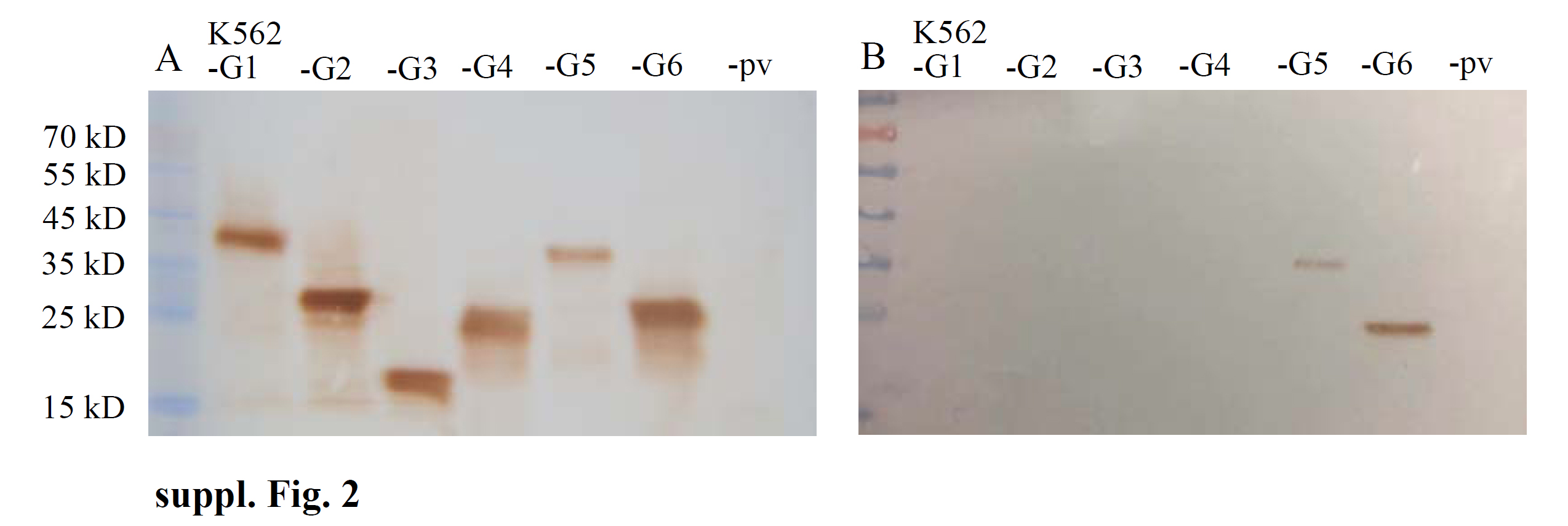

Supplement: Supplementary Figure 2 — Generation of HLA-G1–HLA-G6 isoforms was confirmed by western blotting. (A) HLA-G1–HLA-G6/K562 transfected cell lysates were probed with mAb 4H84 (1:1000, Exbio). (B) HLA-G1–HLA-G6/K562 transfected cell lysates were probed with mAb 5A6G7 (1:1000, Exbio). [file Image2.jpeg]

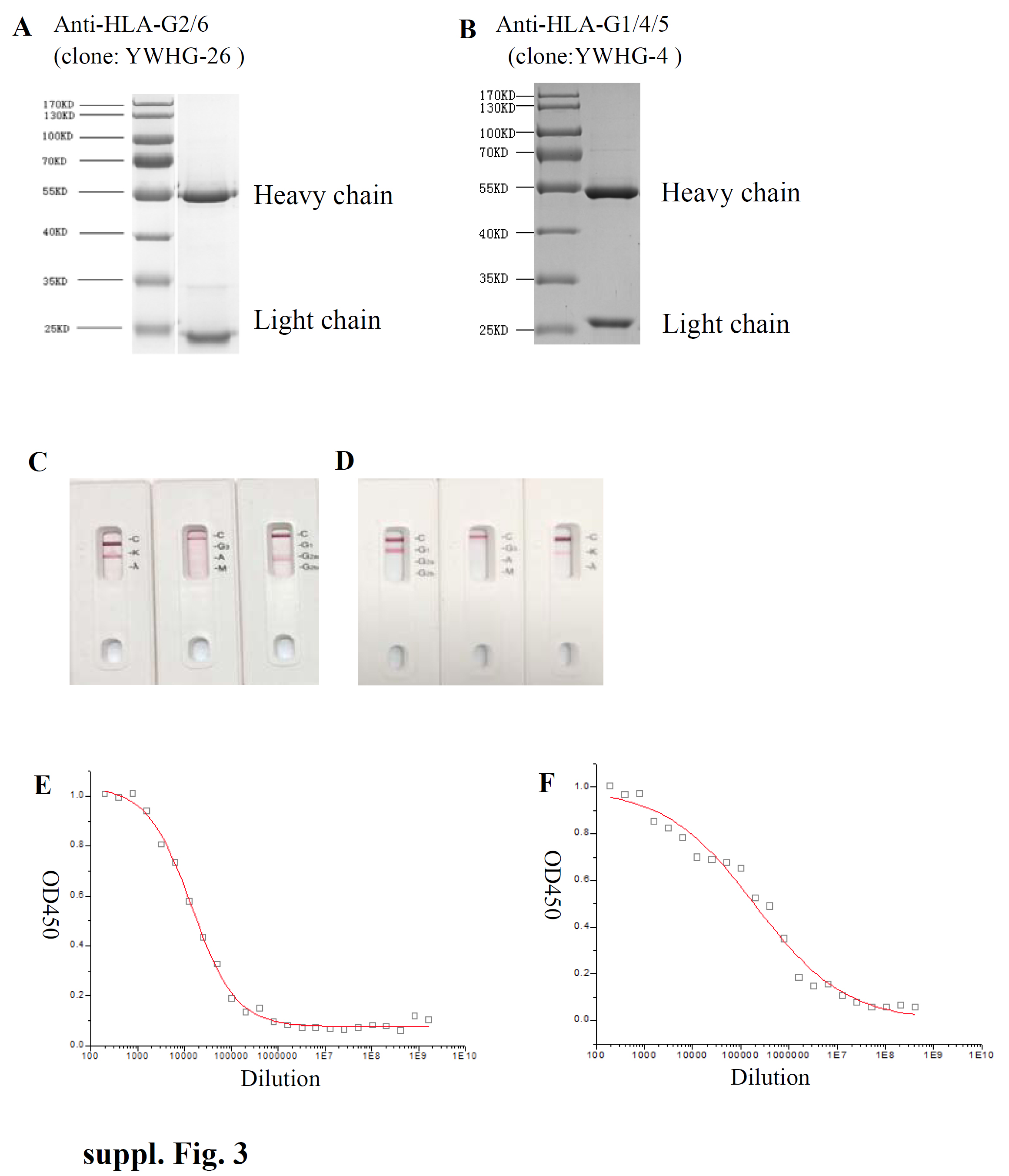

Supplement: Supplementary Figure 3 — Characteristics of the anti-HLA-G2/6 and anti-HLA-G1/4/5 antibodies. SDS-PAGE purity analysis of (A) anti-HLA-G2/6 and (B) anti-HLA-G1/4/5; isotyping of (C) anti-HLA-G2/6 and (D) anti-HLA-G1/4/5; affinity constant curves of (E) anti-HLA-G2/6 and (F) anti-HLA-G1/4/5. [file Image3.jpeg]

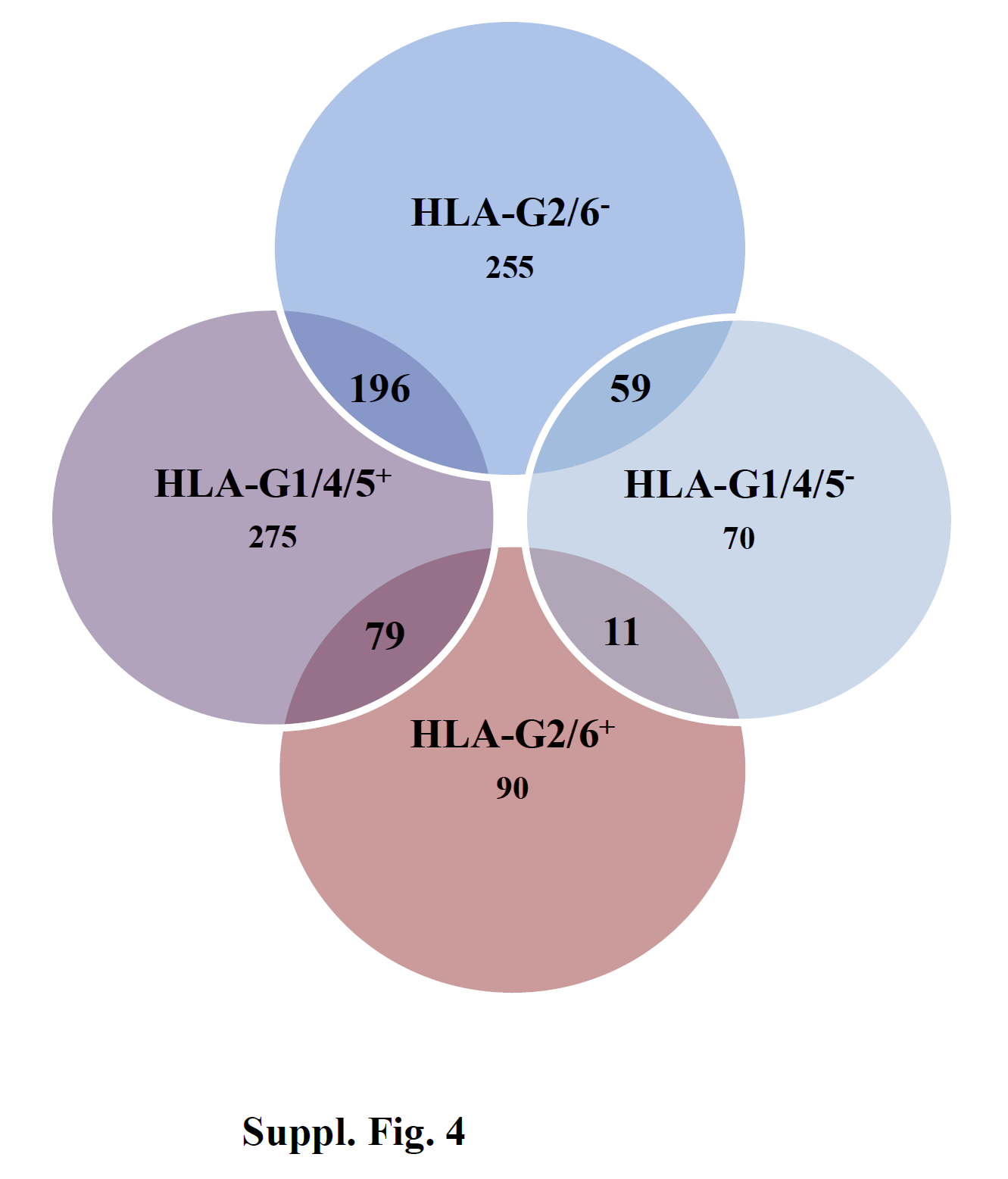

Supplement: Supplementary Figure 4 — Distribution of the co-expression of HLA-G2/6 and HLA-G1/4/5 in patients with CRC. [file Image4.tif]

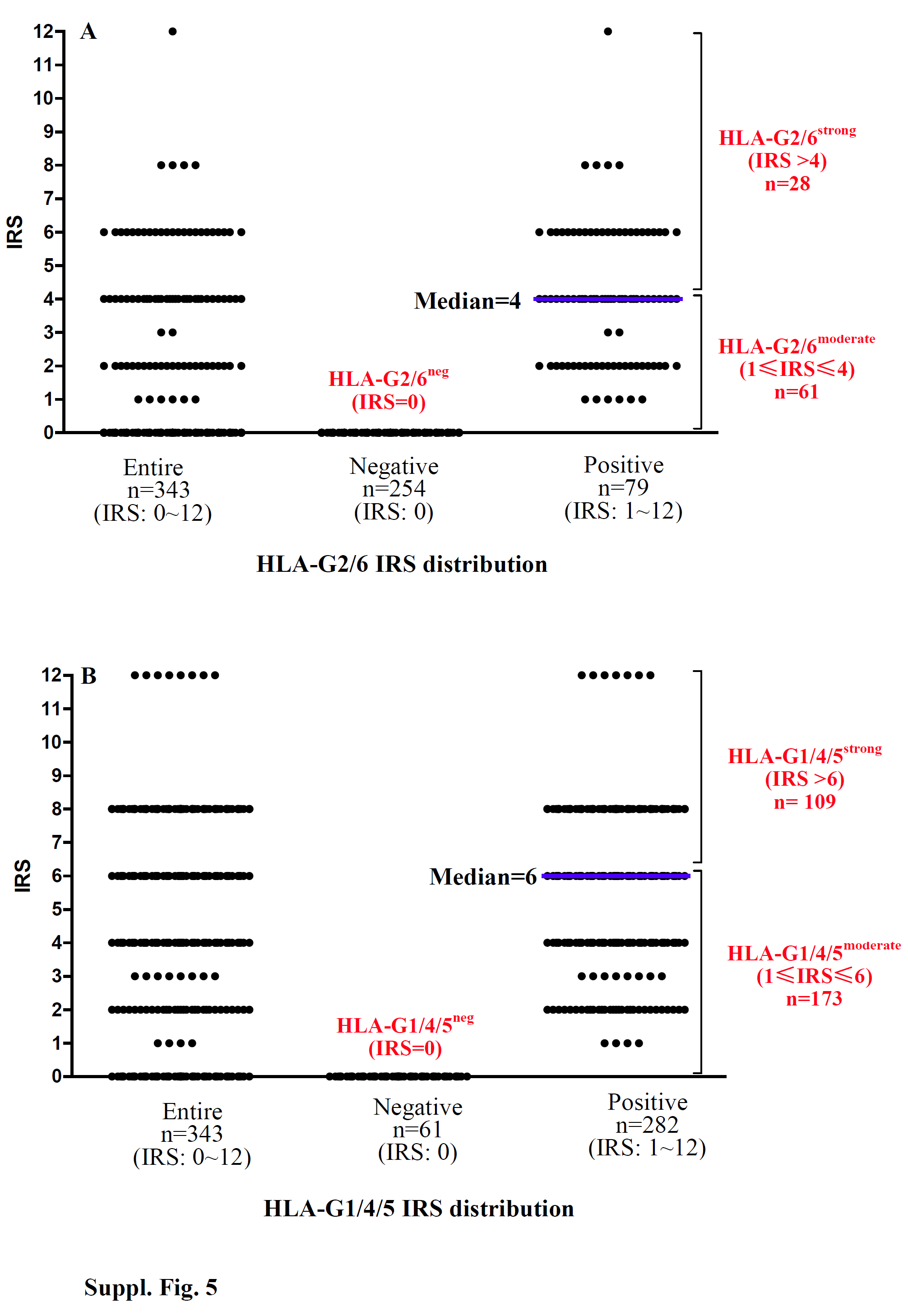

Supplement: Supplementary Figure 5 — Distribution of IRS and grouping (negative, moderate, and strong) of HLA-G2/6 and HLA-G1/4/5 expression in patients with CRC. [file Image5.tif]
