# Supplementary material for: Human leukocyte antigen-G isoform HLA-G2/6, but not HLA-G1/4/5, is an independent indicator of poor survival in patients with colorectal cancer
Source: Front Immunol. 2025 Oct 21;16:1672144. doi: 10.3389/fimmu.2025.1672144 (PMC12583952; doi:10.3389/fimmu.2025.1672144)
Supplement: Supplementary file 6 [file Table1.doc]

| **Suppl. Table 1** RT-PCR primers for HLA-G isoforms | | |
| --- | --- | --- |
| Isoform | Primer sequence (5′- 3′) | Product sizes (bp) |
| HLA-G1 | Sense: TCGA**GAATTC**atggtggtcatggcgccccgaa  Anti-sense: TCGA**CTCGAG**tcaatctgagctcttctt | 1037 |
| HLA-G2 |  | 761 |
| HLA-G3 |  | 485 |
| HLA-G4 |  | 761 |
|  | | |
| HLA-G5 | Sense: TCGA**GAATTC**atggtggtcatggcgccccgaa  Anti-sense: TCGA**CTCGAG**ccaccgaccctgtta | 992 |
| HLA-G6 |  | 716 |
| Note: characters in bold capital represent restriction enzyme site (*EcoR* I and *Xho* I in sense and antisense primer, respectively). | | |
