# Supplementary material for: Human leukocyte antigen-G isoform HLA-G2/6, but not HLA-G1/4/5, is an independent indicator of poor survival in patients with colorectal cancer
Source: Front Immunol. 2025 Oct 21;16:1672144. doi: 10.3389/fimmu.2025.1672144 (PMC12583952; doi:10.3389/fimmu.2025.1672144)
Supplement: Supplementary file 7 [file Table2.doc]

| **suppl. Table 2** Clinical relevance of co-expression status of HLA-G2/6 and HLA-G1/4/5 in CRC patients | | | | | | |
| --- | --- | --- | --- | --- | --- | --- |
| Variables | Cases | HLA-G2/6**^+^**  HLA-G1/4/5**^+^** | HLA-G2/6**^+^**  HLA-G1/4/5**^-^** | HLA-G2/6**^-^**  HLA-G1/4/5**^+^** | HLA-G2/6**^-^**  HLA-G1/4/5**^-^** | *p* |
| Total | 345 | 79 (22.9%) | 11(3.2%) | 196 (56.8%) | 59 (17.1%) |  |
| Sex |  |  |  |  |  |  |
| Male | 204 | 46 | 4 | 122 | 32 | 0.289 |
| Female | 141 | 33 | 7 | 74 | 27 |
| Age |  |  |  |  |  |  |
| ≤ 66 ys | 174 | 42 | 5 | 96 | 31 | 0.615 |
| > 66 ys | 171 | 37 | 6 | 100 | 28 |
| Type |  |  |  |  |  |  |
| Colon | 176 | 43 | 5 | 95 | 33 | 0.663 |
| Rectal | 169 | 36 | 6 | 101 | 26 |
| pT |  |  |  |  |  |  |
| T1+2 | 102 | 24 | 3 | 62 | 13 | 0.560 |
| T3+4 | 243 | 55 | 8 | 134 | 46 |
| pN |  |  |  |  |  |  |
| N0 | 182 | 42 | 6 | 101 | 33 | 0.665 |
| N1 | 95 | 23 | 1 | 54 | 17 |
| N2 | 68 | 14 | 4 | 41 | 9 |
| pM |  |  |  |  |  |  |
| M0 | 339 | 76 | 10 | 194 | 59 | 0.070 |
| M1 | 6 | 3 | 1 | 2 | 0 |
| AJCC stage |  |  |  |  |  |  |
| I | 70 | 19 | 2 | 37 | 12 | 0.942 |
| II | 110 | 22 | 4 | 63 | 21 |
| III+IV | 165 | 38 | 5 | 96 | 26 |
| **^*^** Comparison of HLA-G2/6 and HLA-G1/4/5 expression between or among each variable using the Pearson chi-square test. | | | | | | |
