# Supplementary material for: Human leukocyte antigen-G isoform HLA-G2/6, but not HLA-G1/4/5, is an independent indicator of poor survival in patients with colorectal cancer
Source: Front Immunol. 2025 Oct 21;16:1672144. doi: 10.3389/fimmu.2025.1672144 (PMC12583952; doi:10.3389/fimmu.2025.1672144)
Supplement: Supplementary file 8 [file Table3.doc]

| **suppl. Table 3** Log-rank Mantel-Cox analysis for survival of CRC patients | | | | |
| --- | --- | --- | --- | --- |
| Variables | | Events / No | Survival (95% CI, years) | *p* |
| Sex | Male | 124/203 | 8.8 (7.8~9.7) | 0.024 |
|  | Female | 64/140 | 10.1 (9.0~11.3) |  |
|  |  |  |  |  |
| Age | ≤ 67 ys | 83/172 | 10.1 (9.1~11.0) | 0.010 |
|  | > 67 ys | 105/171 | 8.4 (7.4~9.4) |  |
|  |  |  |  |  |
| Type | Colon | 97/168 | 6.9 (5.0~8.8) | 0.343 |
|  | Rectal | 91/175 | 9.4 (7.7~11.1) |  |
|  |  |  |  |  |
| pT | T1+2 | 54/102 | 10.1 (8.8~11.4) | 0.371 |
|  | T3 | 131/233 | 8.8 (8.0~9.7) |  |
|  | T4 | 3/8 | 10.9 (6.0~15.7） |  |
|  |  |  |  |  |
| pN | N0 | 88/181 | 10.1 (7.0~13.2) | <0.001 |
|  | N1 | 54/94 | 6.1 (4.3~7.9) |  |
|  | N2 | 46/68 | 2.8 (0.5~5.1) |  |
|  |  |  |  |  |
| pM | M0 | 185/338 | 8.7 (7.4~10.3) | 0.751 |
|  | M1 | 3/5 | 4.0 (0.0-8.1) |  |
|  |  |  |  |  |
| AJCC | I | 30/70 | 11.4 (9.9~13.0) | 0.004 |
|  | II | 56/109 | 9.5 (8.0~11.0) |  |
|  | III | 99/159 | 5.5 (4.2~6.8) |  |
|  | IV | 3/5 | 4.0 (0.0-8.1) |  |
|  |  |  |  |  |
| HLA-G2/6 | negative | 127/254 | 10.0 (7.6~12.4) | 0.008 |
|  | positive | 61/89 | 6.3 (4.1~8.5) |  |
|  |  |  |  |  |
| HLA-G1/4/5 | negative | 33/70 | 10.0 (6.4~13.6) | 0.221 |
|  | positive | 155/273 | 7.8 (6.2~9.4) |  |
|  |  |  |  |  |
